# NeuroStream: spectral-spatio-temporal deep learning for visual stimulus classification from EEG

**DOI:** 10.1038/s41598-026-68186-2

**Published:** 2026-09-11

**Authors:** Mohamed Abdelmagid, Marwa Yusuf, Basem M. ElHalawany, Ahmed Fares

**Affiliations:** 1 Electrical Engineering Department, Faculty of Engineering at Shoubra, Benha University, Cairo, Egypt; 2 Present Address: Egypt–Japan University of Science and Technology (E-JUST), Alexandria, Egypt; 3 Computer Science Faculty, Benha National University, Cairo, Egypt; 4 Electronics and Communication Engineering Department, Kuwait College of Science and Technology, Doha District, Kuwait

**Keywords:** EEG-classification, Brain-visual-representations, Multimodal-learning, Deep-learning, Computational biology and bioinformatics, Engineering, Neuroscience

## Abstract

Electroencephalography (EEG)-based visual classification is a challenging task due to low spatial resolution, complex temporal dynamics, and potential experimental confounds, yet with the recent advances in EEG classification, it offers a cost-effective, portable alternative with millisecond-level temporal resolution to Functional Magnetic Resonance Imaging (fMRI) for large scale studies and real-time applications. We propose a novel Spectral-Spatio-Temporal (SST) representation that transforms raw EEG signals into a structured, video-like format. Specifically, we compute wavelet transforms for all channels, aggregate log power into frequency bands, and map these features to electrode positions over time, thereby synthesizing the signal’s multi-dimensional dynamics into a unified, high-fidelity sequence. Building on this representation, we introduce the NeuroStream-SST framework, featuring a lightweight deep learning architecture optimized for spatiotemporal feature extraction. Experiments on the EEGCVPR40 dataset show that our approach reaches $71.25 \pm 0.25\%$ accuracy in the high-gamma band using standard dataset splits, outperforming existing methods evaluated under an identical protocol and demonstrating its ability to capture complex neural characteristics effectively. Furthermore, we implement a set of evaluation protocols designed to expose and quantify the contribution of temporal correlations to reported accuracy. decoding performance declines steadily as the association between class labels and recording sessions is weakened, and falls to the majority-class baseline once the sessions of the evaluated classes are withheld entirely. These findings highlight our framework as a promising direction for EEG-based visual decoding, with implications for brain-computer interfaces and cognitive neuroscience.

## Introduction

Decoding visual information from EEG signals has attracted growing interest due to its potential applications in brain-computer interfaces and cognitive neuroscience^1–5^. Recent studies have demonstrated that visually evoked EEG responses contain discriminative information that can be exploited for image and object classification^6–10^. However, despite steady improvements in reported accuracy, progress in this area remains difficult to interpret. EEG signals are inherently non-stationary, high-dimensional, and strongly subject-dependent, and performance gains are often influenced as much by data representation choices and evaluation protocols as by genuine advances in neural decoding. As a result, it remains unclear which aspects of EEG representations and model architectures are essential for capturing stimulus-related neural activity, and which gains arise from experimental confounds or dataset-specific biases^11,12^.

Early studies achieved remarkable success using fMRI due to its high spatial resolution, enabling detailed mapping of brain regions involved in visual processing^13–16^. However, fMRI is costly, time-consuming, and impractical for real-world applications, constraints that make it prohibitive for large-scale data collection^17^. EEG offers a cost-effective and portable alternative with high temporal resolution, but its low spatial resolution and susceptibility to noise and experimental confounds make visual decoding significantly harder^11,12^. Rather than matching the spatial fidelity of fMRI, EEG’s practical value stems from its scalability and its demonstrated, learnable correspondence with fMRI-derived activity, as evidenced by recent cross-modal EEG-to-fMRI synthesis work^17,18^, which supports its use as a cost-effective modality for large-scale studies and preliminary screening ahead of dedicated fMRI investigations. Recent work has explored machine learning approaches for EEG-based visual classification, leveraging deep architectures to extract spatio-temporal patterns^6–10^.

Despite these efforts, several fundamental challenges remain unresolved. First, many existing approaches treat EEG either as a purely temporal signal or as an unstructured collection of channels^6,7^, overlooking the joint role of spectral dynamics, electrode topology, and temporal evolution. Second, while block design enhances the signal-to-noise ratio by focusing cognitive capacity on the target class^19^, it simultaneously binds class-relevant signals with inevitable temporal correlations, making it difficult to isolate their distinct impacts on reported performance^11,12^. Finally, it remains unclear whether increasingly complex architectures are necessary for this task, or whether compact, task-aware models can capture these multifaceted EEG representations more effectively. To address these gaps, this study is guided by three core research questions: (1) Does incorporating electrode topology alongside spectral and temporal data improve visual decoding? (2) Are video-oriented spatiotemporal architectures more effective than conventional vision models for EEG? (3) To what extent do block-design and subject-specific signatures influence reported performance?

In this work, our main contributions are as follows: (i) We introduce a unified Spectral-Spatio-Temporal EEG representation that encodes frequency content, electrode topology, and temporal dynamics as video-like sequences. (ii) We propose NeuroStream, a lightweight R(2+1)D-based architecture optimized for efficient and interpretable spatiotemporal EEG decoding. (iii) We provide a comprehensive comparison of EEG representations and model families, revealing the advantages of compact, task-specific architectures over large generic vision models. (iv) We present a rigorous evaluation framework that clarifies the impact of block-design and subject-dependent biases on EEG-based visual classification. Together, these contributions offer a principled framework for studying visually evoked EEG representations and provide practical insights into the design and evaluation of robust EEG decoding models.

Throughout this manuscript, *NeuroStream* refers specifically to our core neural network architecture, while *NeuroStream-SST* denotes the comprehensive end-to-end decoding framework combining this architecture with our proposed representation.

## Literature review

Early work on EEG-based visual decoding focused on learning temporal dynamics with recurrent networks. Spampinato et al.^6^ introduced one of the earliest large-scale datasets of visually evoked EEG and reported 82% accuracy with an LSTM^20^-based classifier. Subsequent analysis, however, revealed that low-frequency drifts in the recordings inflated performance, motivating stricter preprocessing and evaluation protocols^7,11^. Building on these insights, Palazzo et al.^7^ proposed EEGChannelNet, a CNN^21^-based model trained in a Siamese, self-supervised manner via contrastive learning to align EEG and image embeddings, reaching an overall accuracy of 60.4% on the filtered data. This approach emphasized learning stimulus-relevant representations rather than session-specific temporal artifacts. Later, Tao et al.^22^ proposed gated transformers for EEG, obtaining 61.11% accuracy with GRU gating on the high-gamma band (55–95 Hz).

Knowledge distillation^23^ has been widely adopted to transfer inductive biases from high-performing models to smaller ones or cross-modal knowledge transfer. CogniNet^24^ used a two-layer LSTM^20^ trained with KL-divergence to mimic visual classifier outputs, reporting improved separability for unseen classes; this suggests enhanced generalization of EEG features to visual semantics. Ferrante, Boccato, Bargione & Toschi^10^ applied cross-modal KD from CLIP^25^ to a CNN^21^ operating on EEG spectrograms, reporting 41.2% accuracy and further demonstrating the feasibility of using powerful vision-language teachers to supervise EEG encoders. To relax the rigidity of logit-matching, relational KD^26^ preserves inter-sample relationships instead of exact outputs; Liu, Dai, Liu, Yi & Kong^9^ distilled from EEGChannelNet^7^ into image models (e.g., ResNet-18^27^, ViT^28^), improving visual classification accuracy (e.g., ResNet-18^27^ from 87.35% to 91.58%), thereby underscoring that EEG-derived relations can inform vision models about brain-aligned structure.

Beyond supervised KD, several studies leverage contrastive learning to align EEG with stimuli. Palazzo et al.^7^ used a Siamese contrastive framework to learn joint EEG-image embeddings. Singh, Dalal, Vashishtha, Miyapuram & Raman^8^ introduced EEGLSTM (60.4% accuracy on filtered EEGCVPR40^6^) alongside EEGCLIP, a training framework that applies contrastive objectives between visual stimuli and corresponding EEG signals. Relatedly, Camaret Ndir, Schirrmeister & Ball^29^ adapted CLIP-like training to align textual information with EEG, showing the broader applicability of contrastive alignment to brain signals.

A parallel line of work pursued architectural rather than supervisory improvements. Schirrmeister et al.^30^ established that deep convolutional pathways on minimally processed signals match expert feature-engineering pipelines, and that factorizing convolution into temporal and spatial stages is an effective inductive bias for EEG. Later architectures made this decomposition multi-scale or multi-branch: EEG-Inception^31^ and EEG-ITNet^32^ process each epoch through parallel branches of different temporal extents, TSception^33^ ties kernel lengths to the sampling rate, and FBCNet^34^ and FBMSNet^35^ obtain multiple scales through filter banks rather than kernels. More recent designs pair convolutional front-ends with attention^36–38^, while EEGNeX^39^ and EEG-SimpleConv^40^ show that carefully tuned purely convolutional networks remain competitive. Together with EEGNet^41^ and ATCNet^42^, these models indicate that parameter efficiency and structured multi-scale processing, rather than depth, drive performance on EEG; we evaluate baselines drawn from these families in Section "Results". Our architecture shares this premise but places the spectral decomposition in the representation, leaving the network to specialize in spatio-temporal processing over topographically faithful maps.

More recently, self-supervised approaches have sought general-purpose EEG representations by pre-training on large unlabeled corpora. BENDR^43^ adapts contrastive masked-prediction objectives from speech modeling to raw EEG, BIOT^44^ tokenizes each channel separately to enable pre-training across datasets with mismatched montages, and LaBraM^45^ scales the idea to roughly 2,500 hours of EEG through a vector-quantized neural tokenizer. These models pursue generality through data volume, whereas we pursue it through an explicit structural prior. The two directions are complementary, as the SST representation defines a structured input space over which such objectives could be applied.

Furthermore, researchers explored architectures and encodings tailored to vision-like processing. Mishra, Raj & Bajwa^46^ transformed raw EEG into grayscale images and used EfficientNet with an SVM classifier, reporting 64% accuracy; treating multi-subject data as multi-channel inputs increased accuracy to 68%, albeit potentially entangling subject-specific cues with stimulus decoding. These methods suggest that spatially organized, image-like or video-like EEG representations may benefit from mature computer vision backbones.

A recurring concern in EEG-based visual classification is the *block design* protocol, where all exemplars of a class are presented consecutively within a session. While this improves signal-to-noise ratio, it introduces session-level temporal correlations that models may exploit, artificially inflating accuracy^6,11^. Subsequent re-analyses of EEGCVPR40^6^ linked early high scores to low-frequency drifts and temporal artifacts^11,12,24^, motivating stricter preprocessing and evaluation. Defenses argue that appropriate filtering and focusing on high-frequency bands can recover cognitively relevant signals^7,19^.

An alternative experimental setup is *randomized trials*, which suppress temporal correlation by interleaving stimuli from different classes^11,47^. However, poor experimental design—such as excessively long sessions—can suppress stimulus responses or cause leakage, as cognitive tasks may persist for several hundred milliseconds^19^. Both designs are widely adopted and present distinct challenges for real-world applications. In this study, we implement complementary strategies to assert the extraction of stimulus-specific responses from EEG collected using block-design experimental setup, including semantic relabeling, session-aware splits, and unseen-class tests.

Collectively, prior work establishes that (i) EEG supports the decoding of visually evoked responses; (ii) contrastive and cross-modal supervision can strengthen alignment with stimulus semantics; and (iii) alternative spatial encodings allow for the adaptation of vision-based architectures. However, existing approaches frequently neglect a unified spectral-spatial-temporal treatment and lack topologically-faithful electrode-to-scalp mappings that evolve over time. These gaps motivate our SST representation. By aggregating wavelet-derived power into spatially mapped video-like sequences, we aim to provide a more comprehensive framework for EEG decoding, supported by a rigorous evaluation strategy designed to expose and quantify the contribution of broader neural correlations to reported performance.

## Methods

### Dataset

In this work, we use the EEGCVPR40^6^ dataset. Spampinato et al.^6^ recorded the EEG evoked from six healthy subjects from homogeneous background while observing a selected subset of images from ImageNet^48^. They used a 128-channel cap with active, low-impedance electrodes (actiCAP 128Ch) with a sampling frequency of 1000 Hz. The images encompassed 40 classes, each represented by 50 samples for a total of 2000 images. Each image was displayed for 0.5 s and all images of the same class were displayed successively followed by 10 s of black screen. The authors divided their experiment for each subject into different sessions to avoid lack of attention. Each session lasted for 350 s, during which subjects viewed the images of ten classes. The authors initially released the raw signal then followed with three versions filtered with the frequency ranges 5–95 Hz, 14–70 Hz, and 55–95 Hz. While each image was displayed for 0.5 s, the authors opted to trim 20 ms from the start and 40 ms from the end to avoid stimulus interference with previous or next image resulting in a 440 ms record of 128 electrodes for each image.

### EEG data representation

While End-to-End learning methods are gaining popularity across the different ML fields, traditional data pre-processing and representation still dominate signal based ML. To enhance the discriminative power of EEG data beyond raw time-series signals, we employed structured representations that capture temporal, spectral, and spatial dynamics relevant to cognitive processing.

#### Time-frequency representation

**Fig. 1:**
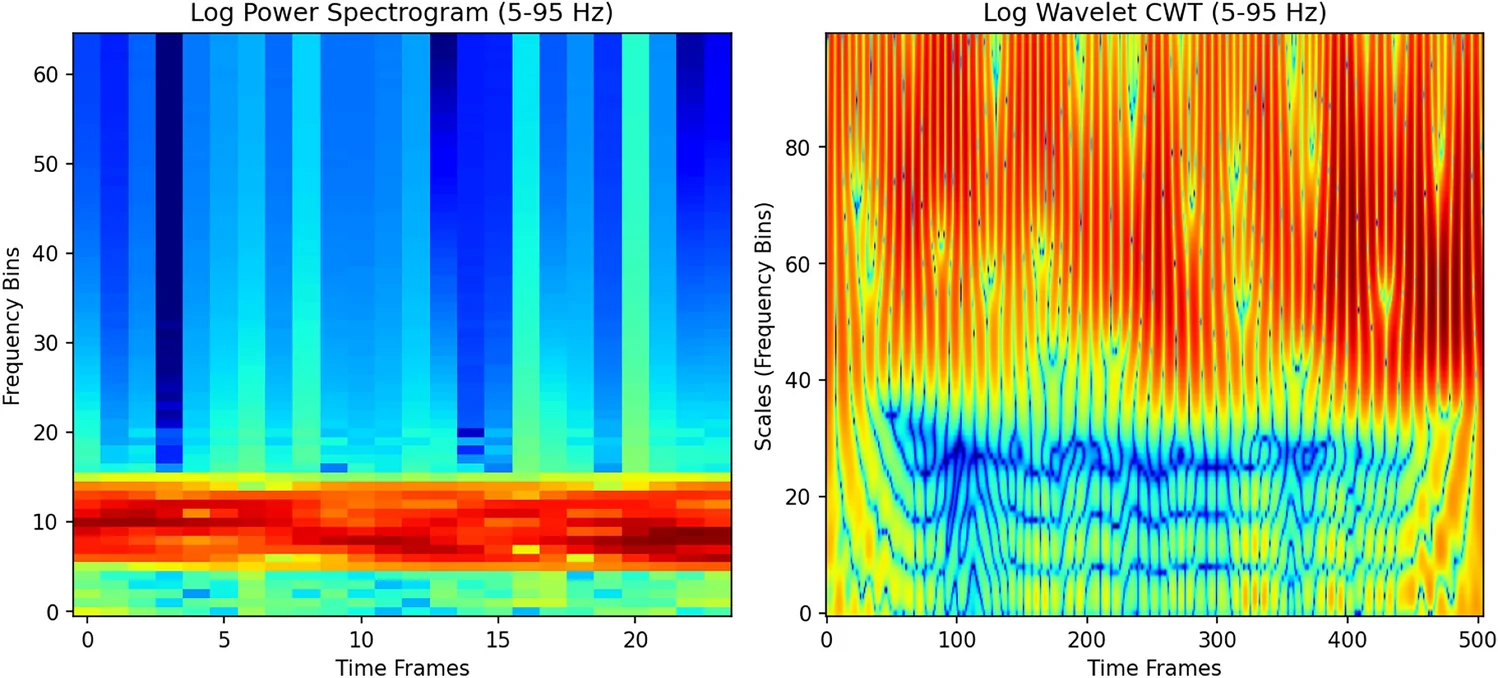
The spectrograms computed with STFT (to the left) and CWT (to the right) for a random signal selected from the 5–95 Hz version of the EEGCVPR40^6^ dataset.

Time-Frequency representations emphasize transient changes across EEG bands that are difficult to extract from raw signals alone purely using neural networks. We computed Spectrograms using Short-Time Fourier Transform (STFT) and Continuous Wavelet Transform (CWT) to provide a joint time-frequency view of brain activity. We configured STFT with a window length and overlap of 128 and 112 samples respectively, covering the frequency range 0–100 Hz, based on preliminary experiments optimizing temporal resolution. For CWT we used the Morlet wavelet with 100 scales spanning 0–100 Hz. Figure 1 illustrates an example spectrogram computed by STFT and wavelet transform for a single channel.

#### Spatiotemporal representation

Electrode positions provide spatial context, as adjacent electrodes often capture activity from overlapping cortical regions. Fares^49^ demonstrated the value of spatial context for EEG classification. Following their approach, we projected the electrode positions onto a 2D 15x11 pixels grid. Each frame encodes the instantaneous amplitude distribution across electrodes. By stacking these frames over time, we generated a video-like sequence that captures both spatial distribution and temporal evolution of brain activity. This representation enables models to learn dynamic activation patterns across cortical regions.

### Spectral-spatio-temporal representation

In this study, we propose a novel multi-dimensional unified EEG representation designed to capture concurrent spectral, spatial, and temporal dynamics. Given an EEG signal $\textbf{X} \in \mathbb {R}^{E \times N}$ recorded across *E* electrodes for a duration of *T* seconds at a sampling rate $F_s$, where $N = F_s \times T$, we transform the raw traces into a spatio-temporal manifold.

To analyze the non-stationary nature of the EEG signal, we first compute a time-frequency representation across all channels using the CWT. While the STFT is efficient for steady-state rhythms, it struggles with the non-stationary nature of EEG due to its fixed resolution, whereas the CWT is superior for capturing both rapid, high-frequency spikes and slow, rhythmic oscillations through its multi-resolution approach. This yields a matrix of size (*E*, *S*, *N*), where *S = 100* represents the number of wavelet scales.

To ensure the representation is robust to the 1/*f* power distribution of EEG signals, we utilize the log-transformed power. We reduce spectral dimensionality by aggregating these values along the scale dimension into *B* discrete frequency bands, each spanning $F_r$ consecutive scales, where $B = S/F_r$. Let $\mathscr {S}_b = \{(b-1)F_r + 1, \dots , bF_r\}$ denote the set of scale indices assigned to band *b*. The average log-power *L* for band *b* at electrode *e* and time sample *n* is defined as:

1 $$\begin{aligned} L_{e, b, n} = \frac{1}{F_r} \sum _{s \in \mathscr {S}_b} \log (|CWT_{e}(s, n)|) \end{aligned}$$

This step yields the average log-spectral density for each band at every discrete time point, effectively condensing the spectral features while preserving the original temporal resolution *N*. Then for each of the *B* frequency bands, we map the average power values from the electrode space *E* to their corresponding 2D coordinates on a topographic grid. This mapping transforms the scalar power values into a spatial frame $\textbf{H} \in \mathbb {R}^{H \times W}$, where *H* and *W* denote the height and width of the spatial grid.

By treating the *B* frequency bands as separate channels (analogous to RGB channels in standard computer vision), we construct a multi-channel topographic image for each time step. Assembled over the total duration *N*, these frames form a spatio-temporal sequence $\textbf{V} \in \mathbb {R}^{N \times B \times H \times W}$. This “video-like” representation encapsulates the evolution of spectral power across the scalp, preserving the spatial context of the sensors alongside their fine-grained temporal dynamics. While this framework utilizes CWT, the representation can be adapted using a STFT. Because STFT naturally produces power estimates within discrete frequency bins, the explicit aggregation step required for CWT is bypassed, allowing for a more direct mapping of frequency bins to the spatial grid.

**Fig. 2:**
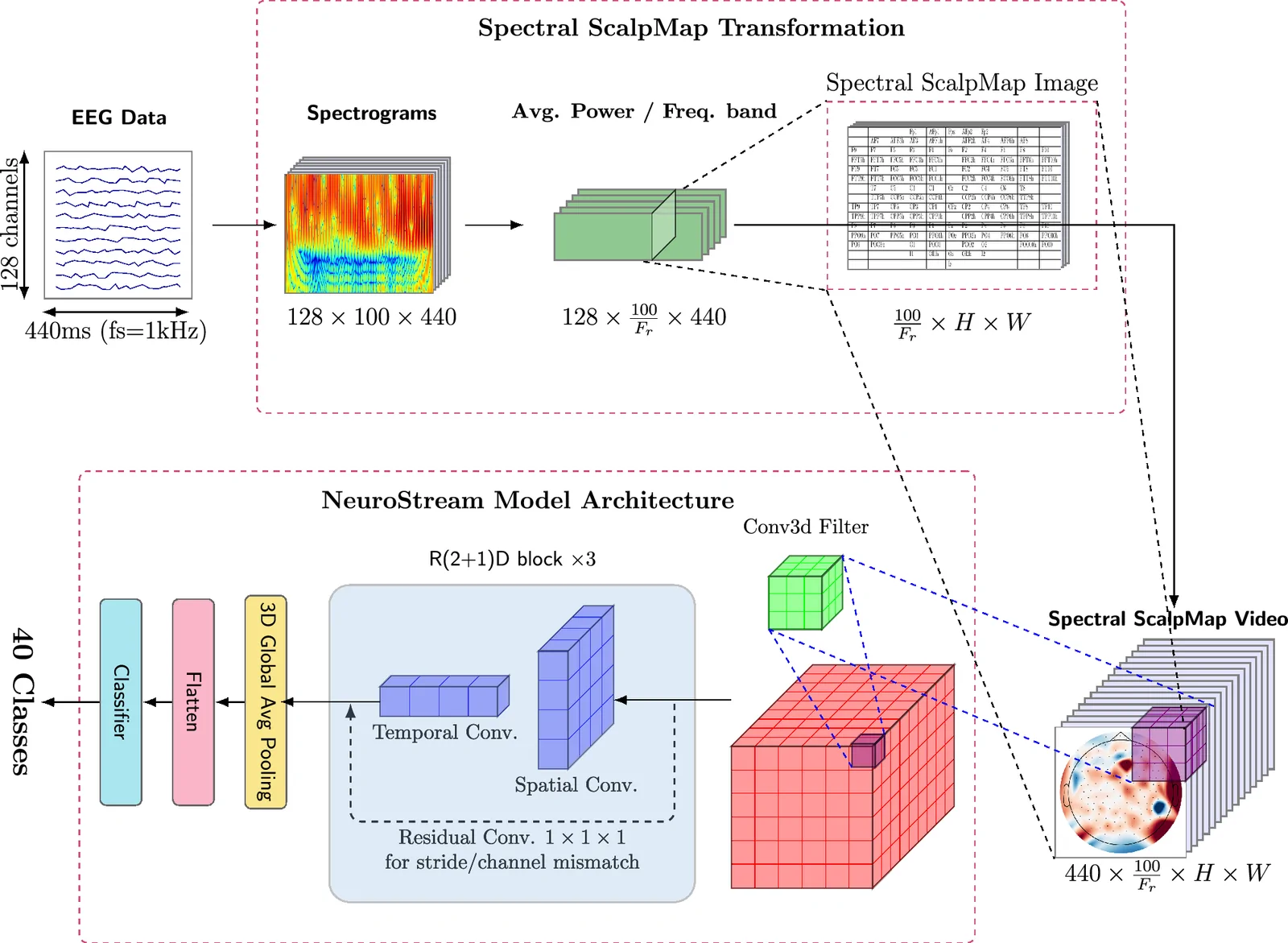
The proposed EEG classification framework: EEG signals are converted into spectrograms, mapped to electrode positions to form spectral scalp maps, and processed by a 3D CNN-based architecture with R(2+1)D blocks, followed by pooling and classification.

### Proposed model

To process the generated Spectral ScalpMap sequences, we propose a novel 3D Convolutional Neural Network (3D-CNN) architecture, termed **NeuroStream**. The model accepts the spatio-temporal input $\textbf{V} \in \mathbb {R}^{N \times B \times H \times W}$ and applies a hierarchical feature extraction process designed to decouple spectral, spatial, and temporal dependencies.

The architecture begins with an initial 3D-CNN layer followed by a series of four **R(2+1)D blocks**. Unlike standard 3D convolutions, the R(2+1)D design factorizes the 3D convolution into a 2D spatial convolution followed by a 1D temporal convolution. This decomposition allows the network to learn spatial electrode relationships and temporal evolution patterns as distinct operations, significantly improving computational efficiency and representation quality while mitigating the risk of overfitting.

Residual connections are integrated across the R(2+1)D blocks to facilitate stable gradient flow and prevent the degradation of low-level spectral information. We employ strided convolutions designed to preserve high-resolution temporal detail while gradually reducing the spatial dimensions (*H*, *W*) of the feature maps.

Following the feature extraction stages, an **adaptive 3D average pooling** layer collapses the spatio-temporal volume into a compact global feature vector. This representation is flattened and passed to a fully connected linear layer for final class prediction. As illustrated in Figure 2, this integrated framework—spanning the Spectral ScalpMap transformation to the NeuroStream—provides a robust and efficient pipeline for decoding complex neural dynamics from multi-channel EEG data.

To justify the structural hyperparameters selected for our framework, we conduct a systematic ablation study, as presented in Section "Results" and summarized in Table 6. Furthermore, the explicit layer-by-layer hyperparameter settings and tensor dimension transformations of this finalized optimal architecture are comprehensively outlined in Table 1.

**Table 1:** Detailed layer-by-layer hyperparameter settings and tensor dimension transformations of the optimal NeuroStream-SST architecture given an input shape of (*B*, 128, 440) where *B* is the batch size.

Operation Type/Sub-Module	Hyperparameters (Kernel, Stride, Padding)	Output Shape $(B \times C \times T \times H \times W)$
Input Data		
Raw EEG Signal	—	$B \times 128 \times 440$
Spectral Mapping		
Log Wavelet Transform	100 Morlet scales, Bin Size = 30, $\epsilon = 10^{-8}$	$B \times 128 \times 4 \times 440$
Dimension Permutation	Permute axes to $(B, \text {Bins}, T, C)$	$B \times 4 \times 440 \times 128$
ScalpMap Projection	2D Azimuthal Mapping Projection (Grid: *15 × 11*)	$B \times 4 \times 440 \times 15 \times 11$
Stem Network		
3D Convolutional Stem	$\text {Conv3D}(4 \rightarrow 48)$, Kernel: (3, 3, 3), Stride: (1, 1, 1), Pad: (1, 1, 1)	$B \times 48 \times 440 \times 15 \times 11$
Batch Normalization & SiLU	BatchNorm3d(48), SiLU()	$B \times 48 \times 440 \times 15 \times 11$
R(2+1)D Blocks		
R2Plus1D_Block (*i=0*)	Temporal Conv: (3, 1, 1), Stride: (1, 1, 1), Pad: (1, 0, 0)	$B \times 48 \times 440 \times 15 \times 11$
	Spatial Conv: (1, 3, 3), Stride: (1, 2, 2), Pad: (0, 1, 1)	$B \times 48 \times 440 \times 8 \times 6$
R2Plus1D_Block (*i=1*)	Temporal Conv: (3, 1, 1), Stride: (2, 1, 1), Pad: (1, 0, 0)	$B \times 96 \times 220 \times 8 \times 6$
	Spatial Conv: (1, 3, 3), Stride: (1, 2, 2), Pad: (0, 1, 1)	$B \times 96 \times 220 \times 4 \times 3$
R2Plus1D_Block (*i=2*)	Temporal Conv: (3, 1, 1), Stride: (1, 1, 1), Pad: (1, 0, 0)	$B \times 96 \times 220 \times 4 \times 3$
	Spatial Conv: (1, 3, 3), Stride: (1, 2, 2), Pad: (0, 1, 1)	$B \times 96 \times 220 \times 2 \times 2$
Classification Head		
Global Pooling	AdaptiveAvgPool3d(1, 1, 1)	$B \times 96 \times 1 \times 1 \times 1$
Flattening	—	*B × 96*
Fully-Connected Layer	Linear(*192 → 40*)	*B × 40*

### Experimental setup

All experiments reported here were conducted on a Nvidia RTX 3060 Ti. for the implementation of models, training, testing, data processing and visualization we used SciPy^50^, SciKitlearn^51^, PyTorch^52^ and Matplotlib^53^. We used the parameters shown in Table 2 and the same dataset split as Spampinato et al.^6^ unless stated otherwise for specific experiments.

**Table 2:** The Experimental hyperparameters used during this study.

Parameter	Value
Learning rate	1e-3
Learning rate decay factor	50%
Learning rate decay every	30 Epochs
Optimizer	Adam
Epochs	200
Batch size	16
Weight decay	None
Dropout	None

No explicit regularization is applied: the model uses neither dropout nor weight decay. Regularization is instead implicit, arising from the compact parameter budget of the architecture, batch normalization after every convolution, the small batch size, the stepwise learning-rate decay, and the selection of the reported checkpoint on validation accuracy.

### Architectural and representation variants

To evaluate the specific contributions of our framework’s components, we introduce targeted variations in our ablation studies. While the default *NeuroStream-SST* framework represents our optimized combination, variations utilizing altered input configurations or alternative feature dimensions are explicitly designated as framework variants (e.g., *NeuroStream-R6*).

### Evaluation protocols

We report **Top-1 accuracy** as primary metric, alongside chance-level baselines (2.5% for 40 classes). For the semantic protocols, whose classes are imbalanced, we additionally report **macro-F1** and **95% confidence intervals** and compare against a majority-class predictor rather than the nominal chance level. For the balanced forty-way task, macro-F1 is redundant with Top-1 accuracy and is omitted.

Each configuration is repeated over random seeds that vary the weight initialization: **three** seeds (13, 42, 87) for the forty-way protocols and **five** (13, 42, 87, 194, 256) for the semantic ones, which are inexpensive to repeat and whose narrow margins over the majority-class baseline demand a tighter estimate of variability. Within a protocol, every model receives the same seeds, splits, preprocessing, and selection rule. Metrics are computed from the confusion matrix of the selected epoch, and we report their mean and standard deviation across seeds.

The forty-way protocols use three partitions: training, validation, and testing. Validation guides training and selects the reported checkpoint, and accuracy is measured on the testing partition alone. The semantic protocols use two partitions only, as the relabeled subset is too small to sustain a third; there the checkpoint is selected at the epoch of highest held-out accuracy and the same partition is reported. The dataset splitting strategy plays a critical role in assessing model performance and ensuring that results reflect stimulus-specific neural decoding rather than experimental artifacts. We adopt multiple evaluation protocols to test generalization under increasingly stringent conditions.

#### Standard split

We use a conventional split of **70% training, 15% validation, and 15% testing**, ensuring that all classes and subjects are represented across all partitions. EEG responses to the same stimulus are kept within the same split to prevent leakage. The chance level for this protocol is **2.5%** (40 classes). Performance is reported using **Top-1 accuracy**.

#### Unseen-class split

To evaluate generalization beyond the training classes, we train the model on **36** classes and hold out **four** classes for testing. After training, EEG features for unseen classes are extracted and evaluated using **SVM classifier**. This protocol tests whether the model learns stimulus-specific representations rather than session-level artifacts.

#### Semantic relabeling

Inherent temporal correlations between proximal EEG recordings are well-documented. To leverage this, Spampinato et al.^6^ utilized a block-design paradigm to induce structured cognitive correlations, thereby minimizing extraneous cognitive load and enhancing the signal-to-noise ratio (SNR) by reinforcing class-specific neural representations. To assess the impact of cognitive correlations on our model’s performance, we implemented a reassignment protocol. We manually re-annotated visual stimuli with ten alternative semantic categories present within the imagery, so that each semantic spans several original classes and is therefore carried by several recording sessions. Samples are divided into an **80/20** split, stratified per semantic and disjoint at the level of the stimulus image, with all six subjects represented on both sides. Because each label now spans several recording blocks rather than a single one, this protocol dilutes the association between the label and any individual session, but it does not remove it entirely; since the original classes still appear in both partitions, so a residual session-level correlation persists.

#### Session-disjoint semantic relabeling

To remove the residual correlation left by relabeling alone, we combine the semantic targets above with a redistribution of the split: **eight** original classes, approximately 20% of the relabeled data, are held out entirely as the test set, so that the recording sessions of the evaluated samples are never seen during training while all ten semantics remain represented on both sides. This is the strictest of our semantic protocols, and contrasting it with the previous one isolates the contribution of within-session temporal correlation to semantic decoding.

#### Session-based split

We divide the dataset such that entire recording sessions are held out during testing to eliminate session-specific temporal patterns. The held-out unit is the subject–class pair rather than the class: each of the **40** classes has the session of exactly **one** subject reserved for testing, distributed as six classes for two of the six subjects and seven classes for each of the remaining four. This yields **40** held-out sessions out of the 240 subject–class recordings, roughly 17% of the data. Every class and every subject is thus represented in training, but never in the combination that is tested, and no session overlaps between the train and test sets. The chance level remains **2.5%** for 40 classes. This protocol tests the model’s ability to generalize across sessions and subjects under strict confound control. All splits, code, and configuration files are publicly available to ensure reproducibility (see the Data Availability Statement and Code Availability sections).

## Results

### Baselines and implementation details

To contextualize the performance of our proposed approach, we compare it against a diverse set of baseline models commonly used for EEG classification. Since several of these architectures share a common design principle, we evaluate one representative per family rather than the full set, covering recurrent models, compact and deep convolutional pathways, inception-style multi-branch designs, filter-bank decompositions, channel attention, and convolution–transformer hybrids:

- **LSTM-based models:** A single-layer LSTM and a four-layer LSTM variant, as proposed by Spampinato et al.^6^.
- **EEGChannelNet**^7^: A CNN based architecture, leveraging temporal and spatial blocks for EEG decoding.
- **EEGNet**^41^: A lightweight CNN based model widely adopted for EEG classification tasks.
- **EEGConformer**^54^: A transformer-based model for EEG signal processing.
- **AttnSleep**^55^: A temporal attention model originally developed for sleep staging. We used the implementation provided by the authors which we previously tested^56^
- **ATCNet**^42^: A state-of-the-art baseline, leveraging multi-head self-attention and temporal CNN blocks to capture complex spatio-temporal dynamics in EEG signals.
- **ShallowConvNet** and **DeepConvNet**^30^: The reference shallow and deep convolutional pathways for EEG decoding, representing the filter-bank-like and the deep-hierarchy ends of the design space.
- **EEG-ITNet**^32^: An inception-style multi-branch architecture combined with dilated causal convolutions for extended temporal context.
- **EEGNeX**^39^: A purely convolutional network obtained by systematically tuning the EEGNet design.
- **EEG-SimpleConv**^40^: A deliberately simple 1D convolutional baseline shown to be competitive with more elaborate architectures on motor imagery.
- **MSVTNet**^36^: A multi-scale convolutional front-end followed by a transformer, designed to capture cross-frequency structure.
- **AttentionBaseNet**^38^: A convolutional backbone augmented with channel attention over electrodes.
- **FBCNet**^34^: A filter-bank network that learns spatial filters per spectral band and aggregates them by variance over time.

For LSTM-1, LSTM-4^6^, EEGChannelNet^7^, and AttnSleep^55^, we utilized the official implementations provided by the respective authors to ensure fidelity to the original logic. For EEGNet^41^, EEGConformer^54^, ATCNet^42^, ShallowConvNet and DeepConvNet^30^, EEG-ITNet^32^, EEGNeX^39^, EEG-SimpleConv^40^, and MSVTNet^36^, we employed the implementations available in the BrainDecode package^57^, a standard library for deep learning in EEG analysis. Every model we trained, including our own, used the same three seeds, splits, preprocessing, and model-selection rule, so those rows are directly comparable; the final two rows of Table 3 are quoted from their original papers and are not comparable on these terms.

We placed particular emphasis on re-evaluating EEGChannelNet^7^ due to significant variance in its reported performance across recent literature. Independent studies have reported accuracies ranging from chance level^12,47^, to 33.5%^10^, 41%^46^, and up to 74.5%^9^.

Table 3 reports the filtered dataset versions only. Earlier single-run experiments on the unfiltered recordings reached 28.89% and 73.5% for the single- and four-layer LSTM^6^, 90% for EEGChannelNet^7^, and 93.09% for AttnSleep^55^. Such accuracies on raw EEGCVPR40 have been attributed to low-frequency drifts and session-level artifacts rather than stimulus-evoked activity^7,11,12^, so we omit them from the table; the drop of 31 to 50 points against each model’s best filtered band indicates how much of that performance the band-pass filtering removes.

Our results, summarized in Table 3, highlight three phenomena. First, every model remains above the 2.5% chance threshold on every filtered band, the weakest entry being the single-layer LSTM at $15.24 \pm 0.80\%$. Filtering therefore does not remove all of the discriminative content of the recordings. What that remaining margin consists of is a separate question, which the protocols of Section "Results" address: performance above chance under this split is not by itself evidence of stimulus-evoked decoding, since the block-design structure leaves session-level correlations available to the models.

Second, the (14–70) Hz band is the weakest version for thirteen of the sixteen models evaluated. The exceptions are the two LSTM variants^6^, which are weakest on the broad (5–95) Hz version, and FBCNet^34^, the only model whose accuracy peaks in the mid band. The mid-band disadvantage is therefore a general pattern rather than an architectural quirk. Architecture-specific preferences emerge across the other two dataset versions: EEGNeX^39^ and ShallowConvNet^30^ peak on the broad (5–95) Hz version, whereas EEGChannelNet^7^, MSVTNet^36^, and EEG-SimpleConv^40^ peak in the high-gamma band. Our framework maintains the highest accuracy on all three versions. Notably, ShallowConvNet exceeds its deeper counterpart^30^ by more than 20 points on the broad band, consistent with our observation that depth alone is not what this task rewards.

Third, the cross-seed standard deviation stays below 3.5 percentage points for every model except two, and at most 1.28 points for ours. The exceptions are EEGChannelNet^7^ on the (14–70) Hz band, reaching 44.56% and 45.01% on two seeds but 20.36% on the third, and the four-layer LSTM^6^ on the (5–95) Hz band, reaching 28.53% and 29.94% against 12.40%; in both cases the spread stems from a single divergent run rather than uniformly noisy training.

Two observations therefore converge on EEGChannelNet^7^. Its accuracy is concentrated in the high-gamma band, 14.4 points above its next-best version, supporting the assertion of Palazzo et al.^58^ that the model relies on high-frequency signals and explaining why preprocessing that suppresses those bands, such as the Supertrials method^59^, disadvantages it disproportionately. Its (14–70) Hz spread of 24.65 points, meanwhile, arises from initialization alone, since the seeds vary nothing else. Initialization is thus sufficient to account for a substantial fraction of the disagreement in the literature noted above, where the same architecture is reported at chance level^12,47^, 33.5%^10^, 41%^46^, and 74.5%^9^, and single-run evaluations of it should be read accordingly.

**Table 3:** Testing accuracy on the standard split of EEGCVPR40^6^ across filtered frequency bands; chance accuracy is 2.5% (40 classes). Macro-F1 is omitted as the forty classes are balanced: it tracked Top-1 accuracy to within 0.65 percentage points on median. Within-run 95% confidence intervals span *± 1.6* to *± 2.2* points, wider than the cross-seed deviations shown.

Model	(5–95) Hz	(14–70) Hz	(55–95) Hz
Reproduced by us under a common protocol (mean ± std over 3 seeds)			
LSTM-1^6^	15.24 ± 0.80%	20.01 ± 0.39%	25.72 ± 0.34%
LSTM-4^6^	23.62 ± 7.96%	37.11 ± 0.89%	42.41 ± 2.03%
EEGChannelNet^7^	38.58 ± 2.85%	36.64 ± 11.51%	52.99 ± 1.10%
AttnSleep^55^	38.93 ± 0.97%	37.92 ± 3.08%	43.11 ± 1.51%
EEGNet^41^	41.38 ± 2.51%	30.78 ± 0.63%	37.92 ± 0.57%
EEGConformer^54^	38.12 ± 3.41%	28.14 ± 0.54%	35.33 ± 1.03%
ATCNet^42^	39.25 ± 0.91%	29.55 ± 1.30%	35.03 ± 0.58%
ShallowConvNet^30^	56.74 ± 0.83%	34.17 ± 1.20%	47.46 ± 0.68%
DeepConvNet^30^	33.65 ± 1.37%	22.24 ± 0.89%	36.69 ± 0.82%
EEG-ITNet^32^	39.63 ± 1.20%	32.04 ± 0.29%	38.22 ± 0.94%
EEGNeX^39^	52.23 ± 1.50%	36.19 ± 2.24%	42.99 ± 0.90%
EEG-SimpleConv^40^	20.61 ± 0.80%	20.18 ± 1.71%	39.84 ± 0.54%
MSVTNet^36^	33.20 ± 2.23%	29.79 ± 0.08%	41.16 ± 1.03%
AttentionBaseNet^38^	46.35 ± 1.52%	36.51 ± 0.48%	43.43 ± 1.26%
FBCNet^34^	32.80 ± 0.64%	34.80 ± 0.61%	30.78 ± 0.24%
**NeuroStream-SST (Ours)**	**68.45 ± 1.28%**	**61.39 ± 0.29%**	**71.25 ± 0.25%**
Reported by the original authors under their own protocols (single values, not directly comparable)			
GRUGate-Transformer^22^	46.42%	47.53%	61.11%
EfficientNet + SVM^46^	64%	51%	–

**Fig. 3:**
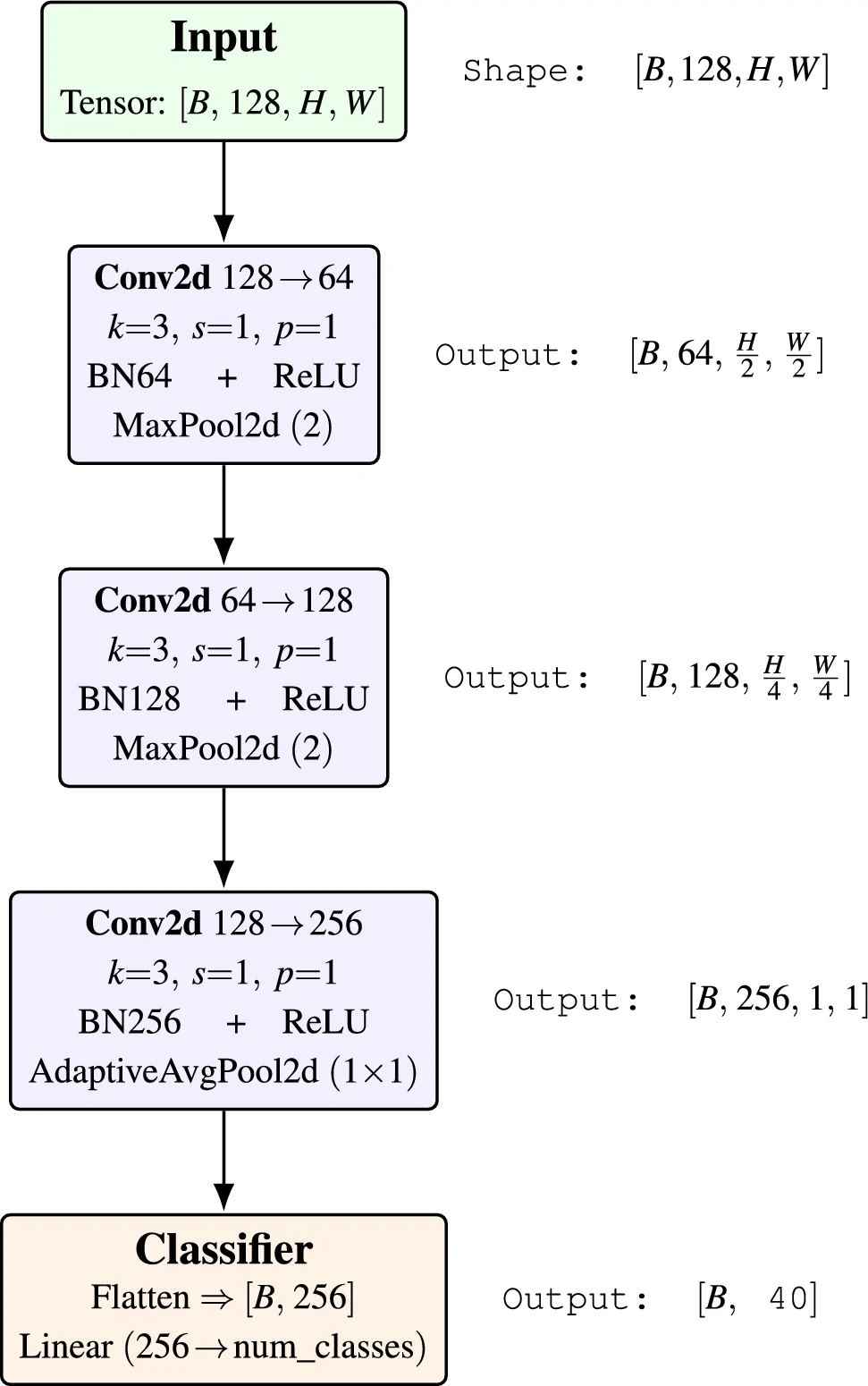
The Architecture of our proposed SpecCNN model.

### Visual representations

Visual representations of neural signals have gained traction due to the availability of robust, pre-trained architectures capable of automated feature extraction from complex data^27,28^. Our proposed SST representation integrates Time-Frequency Spectrograms, which capture localized spectral dynamics, with Scalp Maps that preserve critical spatial topography. Thus we start our study by examining the Time-frequency representations and their contribution for EEG decoding.

To evaluate the efficacy of each input modality, we benchmarked several architectures. For spectral inputs, we utilized ResNet-18^27^, a single-layer Vision Transformer (ViT-1)^28^, and our custom 2D-CNN based model, SpecCNN (Figure 3). For spatio-temporal ScalpMap sequences, we assessed a CNN-TCN hybrid and our NeuroStream model architecture depicted in Figure 2.

The selection of time-frequency parameters is pivotal to model convergence. To optimize the STFT configuration, we conducted a grid search across window sizes $\{32, 64, 128, 224\}$ and overlap ratios $\{50\%, 75\%, 80\%, 90\%\}$. Empirical evaluation indicated that a window size of 128 samples with an 87.5% overlap (112 samples) provided the optimal balance between temporal resolution and frequency binning for this task.

Initial experiments with the ViT architecture—previously successful in sleep staging^56^—yielded results near the 2.5% chance accuracy threshold. We hypothesize that the high parameter density of standard transformers may induce overfitting on the specific noise profile of cognitive EEG data. Subsequent architectural simplification through systematic trials revealed that a shallow configuration (one encoder block with 32 attention heads) was necessary to achieve a peak accuracy of 35.08%. Table 4 provides a comparative summary of these results.

Our findings yield three primary conclusions:

1. **Architectural Parsimony:** Consistent with the results in Table 3, streamlined models like EEGNet and SpecCNN demonstrated superior performance compared to deeper architectures (e.g., ResNet-18^27^, EEGChannelNet^7^), suggesting that for EEG-based classification, domain-specific shallow networks may be more effective than generic deep-vision models.
2. **Feature Resolution:** The choice of signal decomposition method significantly impacts performance. The transition from STFT to CWT-based spectrograms resulted in a substantial accuracy boost, likely due to the CWT’s superior ability to resolve transient, non-stationary cognitive components across multiple scales. Furthermore, despite the simplicity of our SpecCNN model, when using the CWT-based Spectrogram as input, it performed better than most of the baseline models^7,41,54,55^ highlighting the significance of this representation for EEG decoding.
3. **Spatial Integration:** The NeuroStream-R6 model, a lighter version of our model that only leverage spatio-temporal ScalpMaps with no spectral components, achieved overall accuracy (67.08% in the 55–95 Hz band), considerably higher than Spectral representations. The consistent superiority of ScalpMap-based models over pure time-frequency approaches underscores that spatial context is an indispensable feature for high-dimensional cognitive task decoding.

**Table 4:** Testing accuracy on the standard split for selected classifiers applied to the dataset represented as STFT and Wavelet spectrograms and as ScalpMaps. Intended as a heuristic for ranking representations, each entry is a single training run.

Visual Representation	Model	(5–95) Hz	(14–70) Hz	(55–95) Hz
Spectrogram (STFT)	Resnet-18^27^	26.61%	19.66%	32.46%
	SpecCNN	54.84%	37.5%	49.34%
	ViT-1	31.65%	24.85%	31.8%
Spectrogram (CWT)	Resnet-18^27^	41.23%	39.16%	44.46%
	SpecCNN	58.26%	54.49%	57.31%
	ViT-1	35.08%	33.01%	34.12%
ScalpMaps	CNN-TCN	49.75%	52.32%	58.77%
	NeuroStream-R6	65.11%	59.12%	67.08%
Spectral ScalpMaps	NeuroStream-SST	69.86%	60.66%	71.67%

### Performance comparison

In addition to classification accuracy, the practical utility of EEG-based models is largely determined by their computational overhead and inference latency. Table 5 provides a comparative analysis of the evaluated architectures based on two metrics: total number of trainable parameters and average inference time per sample (measured in milliseconds). The evaluation times reported in Table 5 explicitly encompass the entire end-to-end processing pipeline per sample: raw matrix ingestion, GPU-accelerated CWT/STFTs computation, 2D/3D topological projection mapping, and neural network forward pass execution. but does not include preprocessing time condusted by the original authors of the dataset^6^.

An in-depth analysis of the performance metrics reveals a nuanced distinction between a model’s memory footprint (parameter count) and its operational complexity (MACs). While our proposed NeuroStream-SST framework exhibits an exceptionally compact memory footprint—requiring up to two orders of magnitude fewer parameters than heavy backbones like EEGChannelNet^7^ and ResNet-18^27^—it maintains a relatively high computational volume, with $61.64\text {G}$ FLOPs.

This discrepancy indicates high parameter reuse within our architecture, where compact weight layers are repeatedly applied across dense spatio-temporal sequences to maximize feature extraction. Crucially, the data supports our claim that parameter-lightweight, specialized architectures yield superior task-specific performance on EEG data compared to generic, over-parameterized alternatives. Bloated models like STFT_ResNet18 ($11.59\text {M}$ parameters) compress the computational pipeline to $8.81\text {G}$ FLOPs, yet fail to match the specialized decoding capabilities achieved by NeuroStream’s targeted architectural design.

Furthermore, the results demonstrate that the feature representation front-end heavily dictates final pipeline throughput. The choice of input transformation is highly deterministic; for instance, changing the front-end from STFT to CWT on an identical SpecCNN backbone scales the operational load from $2.97\text {G}$ to $78.30\text {G}$ MACs, increasing latency by over *1,400%*. Our NeuroStream variants successfully navigate this trade-off, utilizing a highly optimized weight profile that maximizes accuracy while maintaining real-time inference viability (*<30* ms) on the deployment pipeline.

When cross-referenced with the performance metrics in Table 4, these findings underscore a critical trend: for the EEGCVPR40 dataset^6^, increasing model complexity does not yield proportional gains in accuracy. In fact, more parsimonious models like NeuroStream not only facilitate faster inference but also achieve superior accuracy.

**Table 5:** Comparison of EEG Classification Models in Terms of Parameter Count and Evaluation Time (ms). All benchmarks were evaluated ten times per sample (Batch Size = 1) on an NVIDIA RTX 3060 Ti GPU and average inference time is reported.

Model	Params	FLOPs	Inference Time (ms)
EEGNet	12,024	1.94G	1.100
**NeuroStream-SST (Proposed)**	261,256	61.64G	29.263
NeuroStream-R6	299,080	25.05G	9.655
CWT-SpecCNN	453,992	156.60G	27.276
STFT-SpecCNN	453,992	5.94G	1.751
EEGConformer	571,240	4.53G	10.309
BrainDecoder	1.63M	47.74G	12.305
EEGChannelNet	5.49M	718.84G	75.259
CWT_ResNet18	11.59M	195.72G	27.712
STFT_ResNet18	11.59M	8.81G	6.602

**Table 6:** Ablation study of NeuroStream architectural and representation components, evaluated on the standard split in the (55–95) Hz band. Each configuration is a single training run and *Δ* is measured against C0, so comparisons within the table share one protocol; the seeded result for the full framework is $71.25 \pm 0.25\%$ (Table 3).

Category	ID	Configuration	Accuracy (%)	*Δ*
Baseline	B0	Base model	69.71	1.96
Representation Variations				
	R1	$F_r = 5$	70.26	1.41
	R2	$F_r = 20$	70.49	1.18
	R3	$F_r = 30$	71.09	0.58
	R4	$F_r = 35$	69.10	2.57
	R5	STFT Spectrogram	64.79	6.88
	R6	No Spectral Component	67.08	4.59
Architecture Variations				
	A1	48 Conv3D kernels	71.04	0.63
	A2	48 Conv3D kernels + (3 R(2+1)D blocks)	71.30	0.37
	A3	Reduced depth (2 R(2+1)D blocks)	59.98	11.69
	A4	Increased depth (6 R(2+1)D blocks)	64.89	6.78
	A5	No initial 3D convolution stem	68.67	3.00
	A6	Large 3D convolution kernel ($7\times 7\times 7$)	63.43	8.24
	A7	Spatial-first processing (instead of temporal-first)	69.63	2.04
	A8	No 3D global average pooling	13.26	58.41
Combined Variations				
	C1	64 kernels, 30 aggregated Frequencies, 2 blocks	67.82	3.85
	C2	48 kernels, 25 aggregated Frequencies, 3 blocks	69.18	2.49
	C3	30 aggregated Frequencies, 3 blocks	70.29	1.38
	**C0**	**NeuroStream-SST (Full Proposed Framework)**	**71.67**	**–**

The (55–95) Hz band was selected for ablation as it represents the peak performance of the proposed architecture. The *Δ* column indicates the percentage point drop compared to the *Full* model.

### Ablation study

To empirically validate the design choices of the NeuroStream architecture, we conducted a systematic ablation study focusing on the 55–95 Hz high-gamma band, where the model demonstrated peak sensitivity. We established a baseline configuration (B0) using median parameter values: CWT spectrograms, 10 aggregated frequencies, 32 ($3\times 3\times 3$) Conv3d kernels, and 4 R(2+1)D blocks. We subsequently varied individual components to isolate their impact on decoding accuracy. High-performing variations were then integrated to determine the optimal architectural synthesis. We categorized the study into three primary facets: (1) Representation Ablations, investigating the impact of spectral decomposition methods and frequency aggregation granularity; (2) Architectural Ablations, assessing the influence of the 3D-convolutional layers and number of R(2+1)D blocks; and (3) Combined Ablations, testing the combination of promising parameters. The results, summarized in Table 6, evaluate the contribution of both representation strategies and architectural depth to the overall decoding accuracy.

The results indicate that the method of spectral decomposition is more critical than the granularity of frequency aggregation. Variants R1 and R2 yielded marginal performance declines (1.41% and 1.18%, respectively) compared to the optimal model (C0). This suggests that within the high-gamma band, the architecture is robust to specific frequency binning widths. However, substituting the proposed spectral maps with STFT-based decomposition (R5) resulted in a substantial performance degradation of 6.88%. This drop underscores the superiority of the proposed time-frequency representation in capturing the non-stationary dynamics of EEG signals compared to traditional Fourier-based methods. Furthermore, the complete removal of spectral components (R6) decreased accuracy by 4.59%, confirming that while spatial scalp maps provide a strong baseline, the integration of spectral depth is essential for high-fidelity decoding.

The architectural ablations reveal a high sensitivity to the depth and kernel configuration of the 3D-convolutional pipeline. The most significant performance decline occurred in variant A3, where reducing the depth to two R(2+1)D blocks led to an 11.69% drop in accuracy. This underscores the necessity of a sufficiently deep hierarchical structure to extract complex spatiotemporal features from neural activity. Conversely, increasing the depth to six blocks (A4) was also detrimental (6.78% loss), likely due to overfitting—a common challenge in relatively small-scale EEG datasets like EEGCVPR40^6^.Furthermore, the initial Conv3D layer proved vital for early feature fusion; its removal (A5) resulted in a 3.00% decrease in accuracy. The utilization of large $7 \times 7 \times 7$ kernels (A6) led to an 8.24% performance drop, suggesting that smaller, stacked kernels are more effective at capturing local neural patterns while maintaining a manageable parameter count. The largest drop in the study, however, follows from removing the adaptive 3D average pooling (A8): the flattened tensor then reaching the classifier is 84, 480-dimensional, giving a final linear layer of roughly 3.4M parameters, and the variant memorizes the training set outright, reaching *100%* training accuracy while testing accuracy falls to *13.26%*. The pooled summary therefore does not withhold information the classifier could exploit at this dataset scale, although the variant alters head capacity alongside the pooling itself.

Based on these observations, we identified that the combination of 30 aggregated frequencies, 48 kernels in the Conv3D stem, and 3 R(2+1)D blocks (C0) yielded the highest performance. To investigate potential trade-offs, we tested variants C1–C3 (e.g., substituting kernel density for model depth); however, these configurations consistently underperformed compared to C0. Collectively, these results justify the Full NeuroStream configuration as an optimized architecture for brain representation decoding.

### Bias analysis

To evaluate the reliability and generalizability of the proposed framework, we conducted a comprehensive bias analysis across two primary dimensions: inter-subject variability and class-specific performance. This evaluation aims to identify systematic variations in model performance that may stem from idiosyncratic neural patterns or inherent disparities in stimulus characteristics. By analyzing per-subject accuracies and multi-class confusion matrices, we assess whether the observed performance reflects robust neural decoding of visual stimuli or is significantly influenced by confounding factors such as subject-specific physiological artifacts or class imbalances.

#### Subject-dependent variability

We evaluated the per-subject accuracy of the NeuroStream model on the standard split, in which every participant contributes an equal share of samples to each partition, and then broke the testing partition down by subject. As summarized in Table 7, the model achieved performance significantly above the 2.5% chance threshold for all participants; however, a substantial performance gap was observed between individuals.

For instance, in the high-gamma (55–95 Hz) band, classification accuracy ranged from 62.12% for Subject 1 to 83.18% for Subject 4—a disparity exceeding 21 percentage points. This high degree of subject-dependency aligns with previous findings by Palazzo et al.^7^ and Li et al.^11^, highlighting the “personalized” nature of EEG signals. Given that EEG is frequently utilized for biometric identification^60^, the unique cortical folding and signal-to-noise ratios (SNR) of individual participants present a significant challenge for universal model generalization in small-cohort studies. Notably, the 55–95 Hz band yielded the highest performance for four of the six subjects, with the broad (5–95) Hz version marginally ahead for Subjects 2 and 6, further suggesting that high-frequency oscillations contain the most discriminative features for visual stimulus decoding, largely regardless of individual differences.

**Table 7:** Testing accuracy per subject on the standard split, obtained by partitioning the testing set by subject, for our proposed NeuroStream model trained on data from all subjects.

Subject	(5–95) Hz	(14–70) Hz	(55–95) Hz
1	60.00 %	51.21 %	62.12 %
2	70.78 %	63.55 %	69.88 %
3	73.80 %	73.49 %	74.70 %
4	81.38 %	72.97 %	83.18 %
5	68.48 %	54.55 %	69.39 %
6	67.27 %	53.33 %	66.36 %

**Fig. 4:**
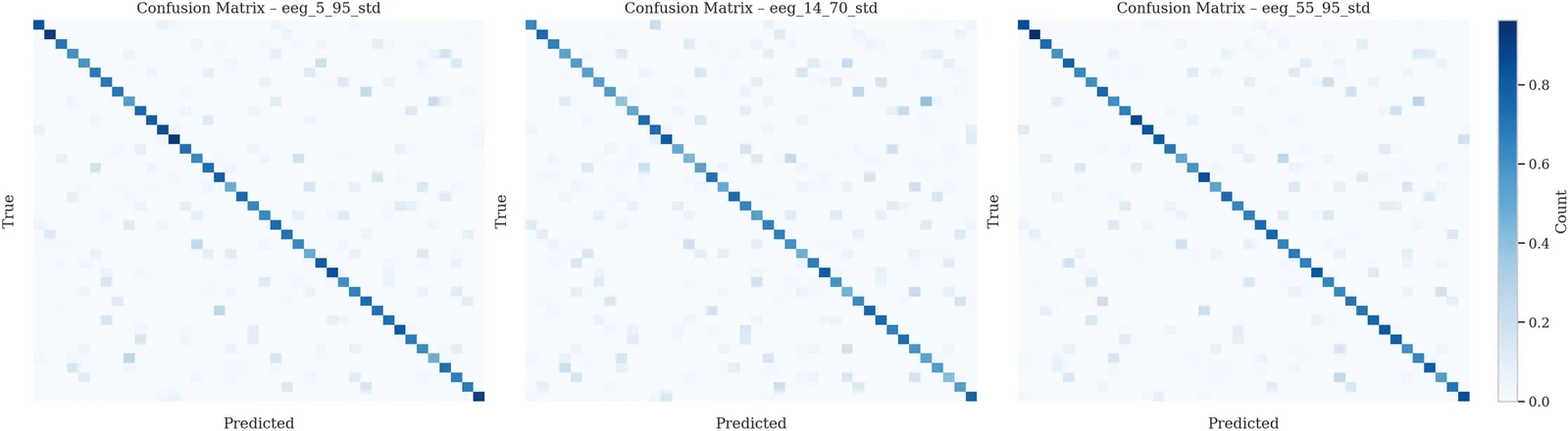
The confusion matrix of our NeuroStream model on the testing partition of the standard split, across the three filtered versions of the dataset.

#### Class-specific dynamics and semantic confounds

To assess class-level bias, we analyzed the confusion matrices (Figure 4) across the three filtered dataset versions. Our analysis reveals both consistent “high-performing” stimuli and recurring misclassifications. Certain categories, such as the *Egyptian cat* (n02124075), *Canoe* (n02951358), and *Parachute* (n03888257), were classified with high precision across all frequency bands, suggesting distinct and stable neural signatures for these specific visual stimuli.

In contrast, several classes were consistently confused, though these errors frequently occurred between semantically related categories. For instance, the *African Elephant* (n02504458) was often misclassified as a *Giant Panda* (n02510455) or *Capuchin Monkey* (n02492035). Similarly, the *Desktop Computer* (n03180011) was frequently confused with the *Computer Keyboard* (n03100240). These patterns highlight shared neural representations or overlapping semantic contexts, indicating that while the model captures high-level categorical information, fine-grained discrimination remains a significant challenge in EEG-based decoding.

### Block design confound

Block design is a long-standing principle of experimental design, introduced to control nuisance variation by grouping observations into homogeneous blocks^61,62^, and it became a standard acquisition paradigm in neuroimaging, where presenting stimuli of the same condition consecutively raises the signal-to-noise ratio of the measured response and has been used extensively in fMRI studies of cognition^63^. The same rationale underlies the acquisition of EEGCVPR40^6^: standard frequency and ocular filtering mitigates environmental artifacts and gross inattentiveness, but the inherent cognitive noise of real-world EEG—unrelated thoughts during the recording—remains, and blocked presentation reinforces the subject’s focus on the target class, occupying cognitive capacity and aligning “background” brain activity with the task at hand. Palazzo et al.^19,58^ argue on this basis that a correctly executed block design remains a legitimate acquisition strategy, and we regard the reinforced state it induces as an authentic, task-relevant signal rather than a pure artifact. It is nevertheless distinct from a direct stimulus-evoked potential, which is precisely the difficulty: accuracy obtained under a block design cannot be attributed to visual decoding alone. To separate these two contributions, we employ the dataset splitting methods detailed in our methodology.

1. **Semantic relabeling.** Following the same approach as Fares^49^, we relabel the stimuli with broader semantics so that a single label spans several original classes and is therefore carried by several recording sessions. This redefines the decoding problem in terms of visual semantics and dilutes the association between the label and any individual session. With the set of semantics available here it does not necessarily eliminate that association, since each semantic still draws on a bounded set of original classes that appear on both sides of the split; a larger and more varied set of semantics would spread every label over correspondingly more sessions.
2. **Session-disjoint semantic relabeling.** Keeping the semantic targets, we additionally withhold whole original classes from training, so the recording sessions of the evaluated samples are never seen. Contrasting this protocol with the previous one exposes the residual session correlation that relabeling alone leaves behind.
3. **Session-based split.** Retaining the original forty-way task, we withhold subject–class sessions rather than classes, so that every class and every subject is seen in training but never in the tested combination. This isolates generalization to unseen sessions of familiar classes and subjects.

**Table 8:** Testing performance on the ten-way semantic task across filtering methods and splitting strategies. Entries are the mean ± standard deviation over five seeds, shared by all models. The 95% CI row is the within-run Wald interval averaged across seeds, which reflects sampling of the evaluation set (*n=432* and *n=640*).

Model	Metric	Raw Signals	(5–95) Hz	(14–70) Hz	(55–95) Hz
Semantic relabeling (majority-class baseline 18.06%, uninformed macro-F1 *≈ 0.10*)					
LSTM^6^	Top-1 (%)	30.23 ± 0.63	24.49 ± 1.19	28.10 ± 0.68	31.81 ± 0.71
	Macro-F1	0.208 ± 0.020	0.138 ± 0.031	0.210 ± 0.020	0.247 ± 0.019
	95% CI (pp)	±4.33	±4.05	±4.24	±4.39
EEGConformer^54^	Top-1 (%)	64.21 ± 0.93	41.48 ± 3.07	44.68 ± 0.80	47.04 ± 1.11
	Macro-F1	0.523 ± 0.012	0.246 ± 0.019	0.239 ± 0.007	0.284 ± 0.010
	95% CI (pp)	±4.52	±4.64	±4.69	±4.71
**NeuroStream-SST (Ours)**	Top-1 (%)	**71.16 ± 0.38**	**52.92 ± 0.28**	**49.81 ± 0.49**	**54.03 ± 0.86**
	Macro-F1	**0.621 ± 0.004**	**0.409 ± 0.007**	**0.364 ± 0.014**	**0.431 ± 0.010**
	95% CI (pp)	±4.27	±4.71	±4.71	±4.70
Session-disjoint semantic relabeling (majority-class baseline 22.94%, uninformed macro-F1 *≈ 0.09*)					
LSTM^6^	Top-1 (%)	20.31 ± 0.90	19.69 ± 0.51	20.16 ± 0.94	20.31 ± 0.88
	Macro-F1	0.102 ± 0.022	0.106 ± 0.010	0.121 ± 0.019	0.104 ± 0.015
	95% CI (pp)	±3.12	±3.08	±3.11	±3.12
EEGConformer^54^	Top-1 (%)	**28.34 ± 1.52**	**24.34 ± 4.02**	**27.19 ± 0.72**	**25.22 ± 1.09**
	Macro-F1	**0.189 ± 0.011**	0.102 ± 0.038	**0.130 ± 0.026**	**0.100 ± 0.018**
	95% CI (pp)	±3.49	±3.31	±3.45	±3.36
**NeuroStream-SST (Ours)**	Top-1 (%)	26.41 ± 1.01	22.19 ± 2.18	21.91 ± 2.37	22.00 ± 1.90
	Macro-F1	0.108 ± 0.036	0.076 ± 0.020	0.084 ± 0.015	0.066 ± 0.018
	95% CI (pp)	±3.41	±3.21	±3.20	±3.20

**Table 9:** Percentage distribution of samples across the ten semantic classes. Semantic relabeling is stratified per semantic, so both of its partitions reproduce the distribution of the entire dataset; only the session-disjoint split departs from it.

Split Type	C1	C2	C3	C4	C5	C6	C7	C8	C9	C10
Entire Dataset	11.00	9.07	15.67	5.50	8.52	12.37	**18.06**	9.90	5.77	4.12
Session-Disjoint Semantic Relabeling										
Train	12.17	8.25	**17.67**	6.28	9.82	9.03	15.97	10.60	5.50	4.71
Test	8.26	11.01	11.01	3.67	5.50	20.18	**22.94**	8.26	6.42	2.75

#### Semantic relabeling and generalization

To evaluate the models’ ability to learn true visual semantics rather than temporal artifacts, we adopted the semantic relabeling approach proposed by Fares^49^. Fares^49^ manually reassigned each image/EEG sample a one of ten semantic labels representing more broad features like dominant color, theme,.etc. Because samples within a single new semantic category originate from different original classes recorded across distinct sessions, this approach dilutes the “block design effect” by making class labels less correlated with specific recording intervals, although, as each semantic is still carried by a bounded set of original classes, the correlation is diluted rather than eliminated. While Fares^49^ utilized an unfiltered version of the dataset, we reconstructed this semantic benchmark using the later-released filtered versions to ensure signal integrity.

Taking the rigor of evaluation a step further, the session-disjoint variant of this protocol isolates a subset of the original classes—representing approximately 20% of the total data volume—to serve exclusively as the test set. In this configuration, the model is trained on a specific set of semantic instances and tested on entirely unseen classes from different sessions. This protocol prevents any leakage of temporal correlations or session-specific noise between the training and test sets, providing the strictest test of whether a model can generalize semantic features to novel experimental contexts. The class distribution of both protocols is markedly imbalanced (Table 9), so the nominal chance level is not a meaningful reference: a predictor that always outputs the most frequent semantic already reaches 18.06% under semantic relabeling and 22.94% under its session-disjoint variant, and label-independent prediction yields a macro-F1 of approximately 0.10 and 0.09, respectively. We therefore read all results against these baselines. We benchmarked three distinct architectures: a baseline LSTM^6^, the hybrid CNN-Transformer EEGConformer^54^, and our proposed NeuroStream-SST. Every model was trained with the same five random seeds under identical preprocessing, splits, and model-selection criteria, so all reported numbers are directly comparable; the reported model is selected on validation accuracy, and Top-1 accuracy, macro-F1, and 95% confidence intervals are computed from the confusion matrix of the selected epoch. Table 8 summarizes the performance of the three models across all filtering versions and splitting strategies.

The three protocols compared here—the standard forty-way split, semantic relabeling, and its session-disjoint variant—differ in both label space and class balance, so we report the baseline-corrected accuracy $\kappa _b = (\textrm{acc} - b)/(1 - b)$, where *b* is the majority-class accuracy on the corresponding test set: 2.50%, 18.06%, and 22.94% respectively. For NeuroStream-SST in the high-gamma band, $\kappa _b$ falls from 0.709 under the standard split to 0.439 under semantic relabeling, a considerable reduction even though the semantic task is nominally easier, with ten broad categories instead of forty and more samples in each. Relabeling therefore suppresses a substantial share of the block-design confound, but not all of it: withholding the recording blocks of the evaluated classes as well drops our model from $54.03 \pm 0.86\%$ to $22.00 \pm 1.90\%$ in the high-gamma band and from $71.16 \pm 0.38\%$ to $26.41 \pm 1.01\%$ on raw signals, with macro-F1 falling to 0.066–0.108, at or below the *≈ 0.09* expected from label-independent prediction. Gaps of this size, 25 to 45 percentage points, exceed both the cross-seed standard deviation (*≤ 4.0* pp) and the within-run confidence interval (*± 3.1* to *± 4.7* pp) by an order of magnitude, and they measure the residual correlation that relabeling leaves behind, each semantic being carried by a bounded set of original classes.

Frequency filtering mirrors this ordering. Raw signals exceed the best filtered band by 31 to 50 points under the forty-way protocol, by roughly 17 points under semantic relabeling, and by at most 4.2 points under the session-disjoint variant, where the advantage reverses for the LSTM^6^. The portion of the raw-data advantage that disappears as session structure is removed is thus better explained by slow, session-linked components than by stimulus-relevant features that filtering discards, which also clarifies the role of filtering: essential under a block design, progressively less consequential once labels are decoupled from individual recording blocks.

Under semantic relabeling, NeuroStream-SST is the strongest of the three models on every dataset version, reaching $71.16 \pm 0.38\%$ on raw signals and $54.03 \pm 0.86\%$ in the high-gamma band, against $64.21 \pm 0.93\%$ and $47.04 \pm 1.11\%$ for EEGConformer^54^ and $30.23 \pm 0.63\%$ and $31.81 \pm 0.71\%$ for the LSTM^6^. Under the session-disjoint variant all three converge toward the majority-class baseline: only the raw-signal configurations exceed it, by $\kappa _b = 0.070$ for EEGConformer and 0.045 for our model, margins that lie within the *± 3.4* pp confidence interval, and only EEGConformer on raw signals reaches a macro-F1 (0.189) clearly above the uninformed value. We therefore treat the session-disjoint protocol as a proof-of-concept probe for a residual stimulus-evoked trace rather than a demonstration of semantic decoding under strict confound control, given that the relabeled subset covers roughly 20% of the dataset and is heavily imbalanced.

#### Unseen classes test

This method was used in other papers working with the same dataset to show generalization on unseen classes^8^. We separated the first 4 classes and trained our model on the rest. We then split the remaining classes into 80-20 split for training and testing so that the EEG responses to a certain image either appeared on the train or test sets but not both. We used only the high gamma dataset version showing best results and lower chance of low frequency noise. The model showed consistent performance on the remaining 36 classes reaching up to 68% classification accuracy. After training, we used the best saved model to extract feature vectors for the segments of the four held-out classes and assessed their separability with an Support Vector Machine (SVM). The pooled feature vectors were shuffled and divided 80/20 at random, without stratification, an Radial Basis Function (RBF) SVM with standardized inputs was fitted on the larger part, and accuracy was measured on the remainder. Against a chance level of 25% for four classes, the classifier reached 99%, indicating that the representation places the unseen classes in near-linearly separable regions of feature space. We note that this split is random at the level of individual segments, so responses of the same subject, and to the same stimulus image, may fall on both sides of it; the test therefore establishes separability within the pooled set rather than generalization to unseen subjects or unseen stimuli.

**Fig. 5:**
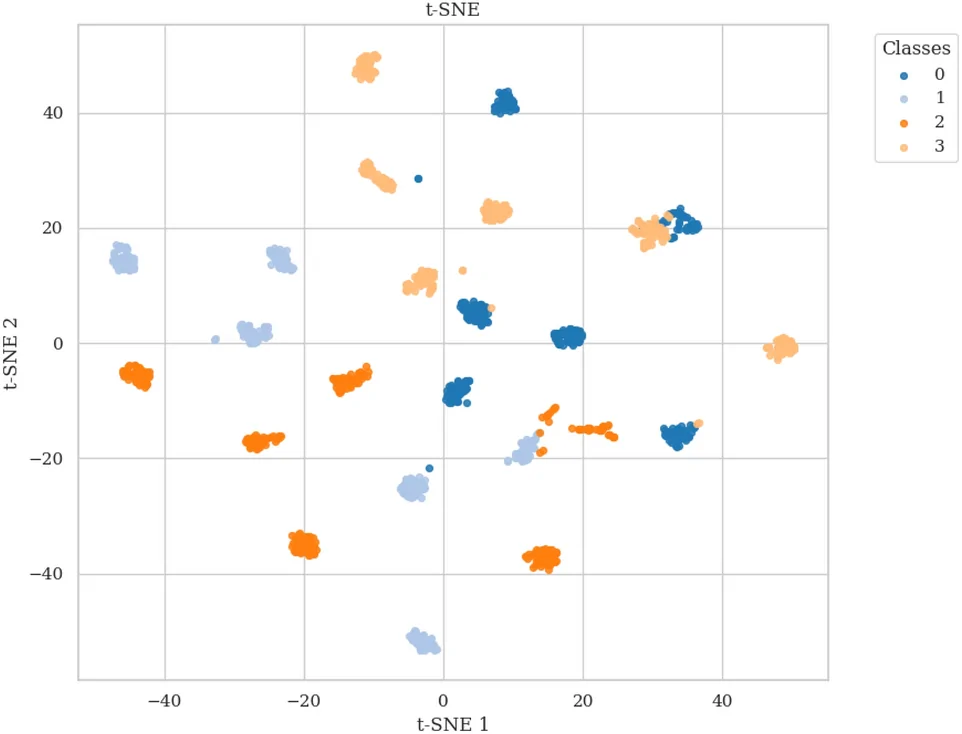
The t-SNE graph of the EEG features of four unseen classes.

Figure 5 shows a visualization of the features extracted corresponding to the remaining four classes. The figure clearly shows that the clusters are visibly separable. However, we also note that each class is split into six visibly differentiable clusters corresponding to each individual subject, suggesting that the cognitive response to stimulus is highly personal in nature and may not be generalized across different subjects.

#### Session-based split

To evaluate the model’s ability to generalize, we adopted a cross-subject session-dropping strategy. For each subject, we removed sessions corresponding to a subset of classes, ensuring that if a session for a given class was dropped for one subject, it remained available for all others. The dropped sessions served as the test set, while the retained sessions were used for training. This design allowed the model to learn from all subjects and all classes, though not necessarily in combination. If the model could identify consistent within-class patterns when presented on a different subject, this would indicate its capacity to generalize across subjects.

Across repeated runs, however, the model achieved less than 1% accuracy on the held-out sessions, below the 2.5% chance level. This outcome suggests that the model relied on subject-specific correlations between classes. This observation can be attributed to two reasons. First, the high flexibility of the model leading to overfitting. Second, The class representation per subject is highly personal and the model could not find a general pattern for each class across all EEG records so it resorted to learn different within-class patterns that led it to learn the individual subject response to each stimulus without being explicitly trained to do so. The second point is further emphasized by the small subject pool.

### Discussion and future work

The high performance reached by our model compared with other approaches using standard data split is highly attributed to the unified representation we used, specially topological information over time which contributes the highest to our performance. However, there is still room for improvement. Our method as is, maintains the relative positions of electrodes but ignores the actual distances between them. We also project the electrode locations over a 2D map while their positions are 3D in reality. While the simplified positioning improves robustness, we believe a more accurate representation can better reflect the cognitive evolution across brain regions more accurately.

Performance declines steadily as we move from the standard forty-way split to semantic relabeling and then to session-disjoint semantic relabeling, and the size of each decline is informative about the confound. Relabeling the stimuli with broader semantics spreads each target label over several recording blocks and reduces baseline-corrected accuracy from 0.709 to 0.439 in the high-gamma band, even though the resulting ten-way task is nominally easier than the original forty-way one. This confirms that a substantial part of the accuracy obtained under the standard split reflects transient brain states induced by the block design rather than purely stimulus-evoked activity. It also shows the limits of relabeling as a remedy: because each semantic is still carried by a limited set of original classes, the label remains partially predictable from the recording session, and withholding the sessions of the evaluated classes brings performance down to the majority-class baseline. Semantic relabeling therefore attenuates the confound rather than removing it, and the two semantic protocols should be read together—the first as a redefinition of the decoding problem in terms of visual semantics, the second as a probe for whatever stimulus-evoked trace survives strict session control. The scope of this experiment was in any case limited to the subset of images and semantic categories proposed by Fares^49^, roughly 20% of the original dataset and heavily imbalanced, which is why we treat its outcome as a proof of concept. Future work will accordingly expand the relabeling effort to a broader array of concurrent semantics and a larger volume of stimuli, so that each semantic spans many more sessions and the residual label–session association is diluted further; at that scale the margins reported here could be resolved rather than merely bounded. Additionally, we intend to conduct cross-dataset validation using the randomized event trials proposed by Ahmed, Wilbur, Bharadwaj & Siskind^47^ to provide a definitive assessment of model robustness against temporal confounds.

The experiments involving unseen classes and dropped sessions yielded critical insights into the personalized nature of neural activity. Our analysis revealed six distinct clusters within each class, each corresponding directly to individual subjects. This clustering suggests that the same stimulus triggers highly divergent cognitive responses across different participants, reinforcing the challenge of intersubject variability in EEG decoding. The following points further validate these observations: (i) Intersubject Divergence: The clear separation of data by subject indicates that the “cognitive fingerprint” of an individual often outweighs the universal neural signature of the class. (ii) Dropped Session Performance: The model achieved near 0% accuracy in the dropped sessions experiment. This result is particularly telling; if the model had successfully decoupled the cognitive response to the stimulus from the subject’s identity (treating them as orthogonal features), we would expect a degree of generalizability. (iii) Correlation of Identity and Response: The failure to generalize across sessions suggests a high degree of correlation between subject identity and cognitive state. This implies that the model learns features that are idiosyncratic to the participant’s internal state during a specific session, rather than invariant features of the stimulus itself.

This collapse under session-based evaluation echoes a broader difficulty documented in the cross-subject and continual EEG decoding literature, where static models trained without explicit adaptation mechanisms suffer substantial performance loss under subject shift^64,65^. Duan et al.^64^ address this via a bi-level meta-learning framework that enables fast adaptation to incoming subjects while retaining knowledge from previous ones, and their subsequent work^65^ mitigates catastrophic forgetting through a stochastic neural-transformation replay mechanism. Rather than expecting a static, single-pass model to learn subject-invariant representations, such online adaptation and replay-based continual learning strategies offer a more principled path toward robust generalization across sessions and subjects, and represent a promising direction for extending the NeuroStream framework.

Blocks design strengthens the SNR by inducing the “expectation of target class”, a class-relevant signal that takes up mental capacity, hence suppressing unrelated cognitive activity. While this method is effective, it does not guarantee the full elimination of unrelated transient activity, raising doubts about how much of the accuracy is derived by visual stimuli. Furthermore, while that signal is class-relevant, it is not specifically visually evoked by definition. On the other hand, randomized trials can eliminate temporal correlations but they can also induce noise through cognitive response leakage across consecutive records. Additionally, the random nature of the experiment requires more subject attention hence the visually evoked signal gets more distorted, leading to the near chance accuracy obtained by previous works and even further demonstrated through our own. Our findings highlight the limitations and risks associated with each experimental design and underscore the need for better experimental design approaches that can enhance SNR without neither block design temporal correlations nor randomized trials stimuli response leakage. Perhaps a hybrid approach –where the stimuli is presented in smaller blocks such that the same stimulus can be presented through multiple blocks– would be better.

While classification based EEG decoding can help extract specific class-relevant patterns from EEG, it has a detrimental weak point: It uses a narrow sighted approach that expects the subjects to focus on the target class in the stimulus image and nothing else, restricting the classification to a very weak signal that might not be present, which motivated the block design reinforcement. Another approach we hypothesize to be more effective for EEG decoding is self supervised contrastive loss. Unrestricted by a target class, this approach reveals the targets of human attention in stimuli images. A model trained with this method would still be able to learn the cognitive correlations in the dataset but it will only matter for the original classes. When coupled with semantic relabeling, this method should have a better chance for generalization.

While these results provide strong evidence within EEGCVPR40^6^, we emphasize that all findings are dataset-dependent. Due to the high subject specificity and block-design structure of EEGCVPR40^6^, generalization to other EEG datasets should be interpreted cautiously. Nevertheless, the observed benefits of spectral-spatial integration and lightweight spatiotemporal modeling are likely transferable to other visually evoked EEG paradigms and EEG classification in general.

## Conclusion

In this study, we addressed the fundamental challenges of visually evoked EEG classification—low spatial resolution and experimental confounds—by introducing a unified SST perspective. By transforming raw EEG into structured, video-like sequences of CWT-based scalp maps, we demonstrated that spectral and topological fidelity are essential for capturing the non-stationary nature of cognitive responses. Our proposed framework, NeuroStream-SST, achieved $71.25 \pm 0.25\%$ accuracy in the high-gamma band (55–95 Hz) across repeated runs, outperforming established state-of-the-art models evaluated under the same protocol on the EEGCVPR40 dataset. Notably, our findings suggest that compact, task-aware architectures are more effective than deeper, generic vision models at learning discriminative EEG patterns while maintaining high computational efficiency. Beyond performance gains, this work provides a critical methodological contribution by rigorously investigating the ’block-design’ confound prevalent in EEG datasets. Through an evaluation framework incorporating session-aware splits, semantic relabeling, and unseen-class testing, we attempted to isolate stimulus-specific decoding from broader temporal correlations. The severe performance degradation observed under session-based splits underscores the persistent challenge of learning subject- and session-invariant representations. While the SST representation preserves high-fidelity features, our findings demonstrate that current architectures remain highly sensitive to temporal and subject-specific blocks, emphasizing the need for robust alignment techniques in future generalizable BCI systems.

## Data Availability

The EEGCVPR40 dataset used in this study is publicly available from the original authors at:https://github.com/perceivelab/eeg_visual_classification. This study does not redistribute the original EEG recordings. The train/validation/test split indices generated for our experiments are publicly available at:https://github.com/aim97/Visual-brain-study/tree/master/resources

## Code availability

The code used to preprocess the data and implement the Spectral–Spatio–Temporal (SST) model is available in our project repository:https://github.com/aim97/Visual-brain-study.
